# Prognostic impact of muscle mass in idiopathic interstitial pneumonia: analysis of idiopathic pulmonary fibrosis and other idiopathic interstitial pneumonias

**DOI:** 10.1186/s12890-025-03942-0

**Published:** 2025-10-14

**Authors:** Hirotaka Hagiwara, Tomotsugu Takano, Hiroaki Ogata, Kazuya Tsubouchi, Katsuyuki Ichiki, Shohei Takata, Hiroshi Ishii, Yasuhiko Kitasato, Yoshiaki Zaizen, Kazuhiro Yatera, Masayuki Kawasaki, Masaki Fujita, Makoto Yoshida, Takashige Maeyama, Ayano Mashimoto, Kazuto Furuyama, Ryo Torii, Kunihiro Suzuki, Yuichi Mizuta, Kazunori Tobino, Eiji Harada, Fumiaki Kiyomi, Hidetake Yabuuchi, Yoichi Nakanishi, Isamu Okamoto

**Affiliations:** 1 Department of Respiratory Medicine, Graduate School of Medical Sciences, Kyushu University, Fukuoka, Japan; 2 Kirigaoka Tsuda Hospital, Kitakyushu, Japan; 3 Department of Respiratory Diseases, NHO Fukuoka Higashi Medical Center, Fukuoka, Japan; 4 Department of Respiratory Medicine, Fukuoka University Chikushi Hospital, Chikushino, Japan; 5 Department of Respiratory Medicine, Japan Community Health Care Organization Kurume General Hospital, Kurume, Japan; 6 Division of Respirology, Neurology, and Rheumatology, Department of Medicine, Kurume University School of Medicine, Kurume, Japan; 7 Department of Respiratory Medicine, School of Medicine, University of Occupational and Environmental Health, Kitakyushu, Japan; 8 Department of Respiratory Diseases, NHO Omuta National Hospital, Omuta, Japan; 9 Department of Respiratory Medicine, Fukuoka University School of Medicine, Fukuoka, Japan; 10 Department of Respiratory Diseases, NHO Fukuoka National Hospital, Fukuoka, Japan; 11 Department of Respiratory Medicine, Hamanomachi Hospital, Fukuoka, Japan; 12 Department of Respiratory Medicine, Japan Community Health Care Organization Kyushu Hospital, Kitakyushu, Japan; 13 Department of Respiratory Medicine, Saiseikai Fukuoka General Hospital, Fukuoka, Japan; 14 Department of Respiratory Medicine, Wakamatsu Hospital of the University of Occupational and Environmental Health, Kitakyushu, Japan; 15 Department of Respiratory Medicine, Steel Memorial Yawata Hospital, Kitakyushu, Japan; 16 Department of Respiratory Medicine, St. Mary’s Hospital, Kurume, Japan; 17 Division of Respiratory Medicine, Aso Iizuka Hospital, Iizuka, Japan; 18 Department of Respiratory Medicine, Kitakyushu Municipal Medical Center, Kitakyushu, Japan; 19 Clinical Research Support Center Kyushu, Fukuoka, Japan; 20 Department of Health Sciences, Graduate School of Medical Sciences, Kyushu University, Fukuoka, Japan; 21 Kitakyushu City Hospital Organization, Kitakyushu, Japan

**Keywords:** 5-year survival rate, Real-world registry, Idiopathic interstitial pneumonia, Muscle mass, Acute exacerbation

## Abstract

**Background:**

Low skeletal muscle mass has been reported to associated with poor prognosis in patients with idiopathic pulmonary fibrosis (IPF). However, such associations have scarcely reported in idiopathic interstitial pneumonias (IIPs) other than IPF. Quantification of muscle mass obtained from chest computed tomography (CT) is used as a simple screening tool for sarcopenia in patients with respiratory diseases. However, the optimal thoracic site for muscle mass quantification is controversial. Moreover, there have been no reports investigating the association between muscle mass and acute exacerbations. This study aimed to evaluate optimal site for muscle mass quantification in chest CT to predict survival and acute exacerbation in IPF and non-IPF idiopathic interstitial pneumonias.

**Methods:**

This study included 528 patients diagnosed with IIP at 29 facilities between September 1, 2013, and April 30, 2016, following multidisciplinary discussions with prospective follow-up over a 5-year period. The cohort was divided into two groups: those with IPF and those with non-IPF IIPs. Skeletal muscle mass was quantified using the erector spinae muscle index (ESMI) and pectoralis muscle index (PMI), defined as the respective muscle area on chest computed tomography (CT) divided by height squared at the time of enrollment. Associations between these indices at baseline and both survival and acute exacerbation were analyzed.

**Results:**

In both IPF and non-IPF cohorts, Cox regression analysis revealed that patients with low ESMI had a poorer prognosis compared to those with normal ESMI, even after adjusting for age, sex, % forced vital capacity (FVC), and smoking exposure level. The hazard ratios were 0.62 (95% CI 0.40–0.90; *p* = 0.013) and 0.46 (95% CI 0.26–0.83; *p* = 0.009), respectively. In contrast, no significant relationship was identified between PMI and survival. Multivariable Cox regression analysis confirmed that ESMI was an independent predictor of survival in both IPF and non-IPF patients. Additionally, acute exacerbations occurred more frequently in the low ESMI group and low PMI group, particularly among non-IPF patients.

**Conclusions:**

The ESMI obtained from chest CT is associated with survival in not only in IPF patients but also in the non-IPF patients. The ESMI and PMI also associate with acute exacerbations in non-IPF patients.

**Supplementary Information:**

The online version contains supplementary material available at 10.1186/s12890-025-03942-0.

## Background

Idiopathic pulmonary fibrosis (IPF) is a chronic progressive lung disease with an average reported prognosis of approximately 3 to 5 years following diagnosis [1, 2]. Among interstitial lung diseases (ILDs) other than IPF, 15–40% of cases exhibit fibrosis progression, with a prognosis as poor as that of IPF, highlighting the importance of cross-disease phenotypic intervention [3–5]. Given the progressive nature of ILDs, identifying key interventional features and potential preventive strategies is needed [6].

Patients with IPF frequently experience reduced physical activity and exercise capacity due to impaired lung function.

Sarcopenia is defined as a condition characterized by a decline in muscle strength, skeletal muscle mass, and physical function, according to the AWGS criteria [7]. Sarcopenia has been reported to be present in 23–39% of patients with IPF [8] and is associated with hospitalizations and mortality in chronic respiratory diseases [9, 10]. In recent years, several studies have highlighted the association between low skeletal muscle mass and poor prognosis in COPD, lung cancer, and pulmonary Mycobacterium avium complex infection [11–15]. Recent studies have demonstrated that skeletal muscle mass quantification of the pectoralis muscle and erector spinae muscle on routine chest computed tomography (CT) offers a simple, practical surrogate for evaluating sarcopenia in patients with respiratory diseases without additional radiation exposure. Moreover, lower CT-derived muscle mass has been associated with poorer survival in idiopathic pulmonary fibrosis IPF [16–20]. However, it remains unclear which specific muscle mass measure may more accurately predict prognosis. Furthermore, most reports have been retrospective, single-center studies focused on survival outcomes, and evidence concerning ILDs other than IPF remains limited, aside from one study on idiopathic pleuroparenchymal fibroelastosis (iPPFE) [11]. Moreover, there have been no reports investigating the association between muscle mass and acute exacerbations. This study aimed to evaluate whether the cross-sectional area of the pectoralis muscle at the fourth thoracic vertebra (T4) level and the cross-sectional area of the erector spinae muscle at the twelfth thoracic vertebra (T12) level are associated with survival and acute exacerbation in IPF and non-IPF idiopathic interstitial pneumonias. The analysis utilized a multicenter cohort of patients diagnosed through multidisciplinary discussion (MDD) and prospectively followed for survival and acute exacerbations over 5 years. Additionally, we examined the relationship between muscle mass, pulmonary function, and subjective symptoms at the time of enrollment.

## Methods

### Study design

This was a prospective and multicenter observational study of patients with IIPs from 29 centers who were followed up longitudinally for 5 years.

### Study patients

**Fig. 1:**
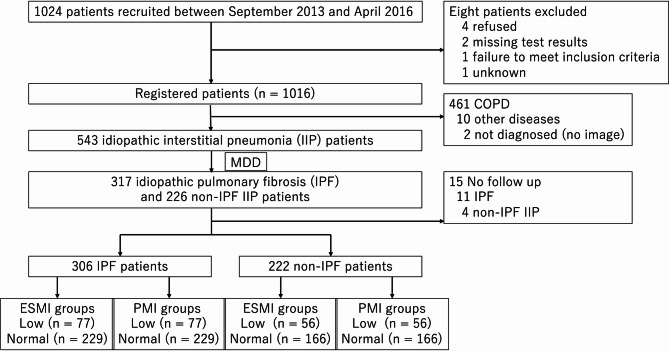
Flow diagram for the study COPD, chronic obstructive lung disease; IPF, idiopathic pulmonary fibrosis; MDD, multidisciplinary discussion; ESMI, erector spinae muscle index; PMI, pectoralis muscle index

The Tobacco-Related Lung Disease Registry Study Group in Fukuoka collected data from 29 facilities on patients aged 20 and older with IIPs and chronic obstructive pulmonary disease (COPD). Patients were included regardless of prior diagnoses, while those with concomitant conditions such as heart failure, infectious pneumonia, pulmonary complications, co-existing lung cancer, or other uncontrolled malignancies were excluded. Patients were recruited from participating facilities in Fukuoka Prefecture. A total of 1,024 cases were collected between September 1, 2013, and April 30, 2016. Eight cases were excluded (four due to consent withdrawal, two due to missing test results, one deemed ineligible, and one unspecified), resulting in 1,016 participants. Following enrollment, the central diagnosis committee re-evaluated the diagnoses of IIPs and COPD. Ten cases with alternative diagnoses and two cases classified as undiagnosable were excluded, leaving 543 cases diagnosed with IIPs and 461 cases diagnosed with COPD. The diagnosis of interstitial pneumonia was made according to the American Thoracic Society/European Respiratory Society/Japanese Respiratory Society/Latin American Thoracic Association (ATS/ERS/JRS/ALAT) IPF 2011 guidelines and the ATS/ERS IIPs 2013 guidelines, through an MDD involving pulmonologists, radiologists, and pathologists [2, 21, 22]. The collected clinical data included information on age, sex, symptoms, medical history, smoking history, laboratory tests, pulmonary function tests, and CT imaging [2, 23]. Follow-up surveillance was conducted annually for 5 years to evaluate exacerbation and death. Exacerbation was determined by a clinician based on international criteria [22]. All examinations and investigations were performed as part of routine care for each patient at the physician’s discretion, and no additional visits or investigations were mandated for this study. A survival survey was conducted with a 5-year longitudinal follow-up. Among the 543 enrolled patients with IIPs, 15 cases without follow-up data were excluded, leaving a total of 528 patients for analysis (Fig.1).

### Pulmonary function tests and CT

Predicted forced expiratory volume in 1 s (FEV1), vital capacity (VC), and forced vital capacity (FVC) were calculated using a reference equation reported by the Japanese Respiratory Society (JRS) in 2001 [24]. The cross-sectional area of the PM at the level of the T4 and the cross-sectional area of the ESM at the level of the T12 were manually marked and measured using Slicer 5.6.2 (http://www.slicer.org) on 5-mm collimation CT scans obtained at the time of participant registration (Figure S1). The erector spinae muscle index (ESMI) and pectoralis muscle index (PMI) were calculated by adjusting the measured muscle areas for the patients’ heights (PMI: pectoralis muscle area/height^2^, ESMI: erector spinae muscle area/height^2^) [18, 25]. In the absence of established reference values for ESMI and PMI, participants were categorized based on quartile distribution, as described in previous studies [16, 17]. Patients in the upper three quartiles of ESMI values were defined as having normal ESMI, while those in the lowest quartile were defined as having low ESMI. Similarly, normal PMI and low PMI were defined using the same quartile-based approach.

### Informed consent

This prospective, multicenter, observational study was approved by the Institutional Review Board of Kyushu University (approval numbers: #25–135 [August 23, 2013] and #555-00 [August 27, 2013]), as well as by the Institutional Review Boards of all participating hospitals, including: Kirigaoka Tsuda Hospital (#5); NHO Fukuoka Higashi Medical Center (#25-2-4); Fukuoka University Chikushi Hospital (#R13-030); Japan Community Health Care Organization Kurume General Hospital (#133); Kurume University School of Medicine (#168); University of Occupational and Environmental Health (#H25-108); NHO Omuta National Hospital (#25 − 20); Fukuoka University School of Medicine (#13-11-06); NHO Fukuoka National Hospital (#25 − 21); Hamanomachi Hospital (#2013-26); Japan Community Health Care Organization Kyushu Hospital (#276); Steel Memorial Yawata Hospital (#13–54); St. Mary Hospital (#13-1019); Aso Iizuka Hospital (#25–189); Fukuoka Sanno Hospital (#FS-81); Kyushu Central Hospital (#62); NHO Kokura Medical Center (#141); Kyushu Rosai Hospital (#15-7-1); and Japanese Red Cross Fukuoka Hospital (#250).In addition, the following three institutions—Japan Community Health Care Organization Fukuoka Yutaka Central Hospital, Nishifukuoka Hospital, and Saiseikai Iizuka Kaho Hospital—were reviewed and approved by the Central Institutional Review Board of the Clinical Trial Network Fukuoka (approval number: #13-E14). For institutions where approval numbers are not officially assigned according to their regulations, a copy of the original approval document was submitted to the journal. All the ethics committees involved in this study are based in Japan. Written informed consent was obtained from all patients.

### Patient and public involvement

Patients or the public were not involved in the design, or conduct, or reporting, or dissemination plans of our research.

### Statistical analysis

R software version 4.4.0 (R Foundation for Statistical Computing, Vienna, Austria) was used to perform all statistical analyses. Two-sided *p* < 0.05 was considered to indicate statistical significance. For baseline characteristics, the heterogeneity in each variable between the groups was evaluated using the Student’s t -test, chi-square test, or Mann-Whitney U test. Kaplan–Meier curves were constructed to show the overall survival of the ESMI groups and PMI groups. The influence of ESMI and PMI on all-cause mortality was estimated as hazard ratios (HRs) with 95% confidence intervals (95% CIs) using simple and multivariable Cox proportional hazard models. The multivariable models included age, sex, %FVC, and smoking exposure level as covariates. BMI was significant in univariate survival analysis only in the IPF cohort; its inclusion in the Cox proportional hazards models did not improve model fit in both cohorts. Consequently, BMI was not incorporated as an adjustment covariate in the multivariable analyses.

In the multivariable-adjusted Cox proportional hazards analysis, all the aforementioned potential confounders were used, except for brain natriuretic peptide (BNP), N-terminal proBNP, surfactant protein-D (SP-D), surfactant protein-A (SP-A), and modified Medical Research Council (mMRC) score, which were excluded due to a high number of missing values. The influence of ESMI and PMI on acute exacerbation was estimated as HRs with 95% CIs using simple and multivariable Cox proportional hazard models. The multivariable models included age, sex, %FVC, and smoking exposure level as covariates. The correlation between ESMI or PMI and %FVC was analyzed using Spearman’s rank correlation test. The association between mMRC score and either ESMI or PMI was assessed using an ordinal logistic regression model.

## Results

### Study subjects

A total of 528 patients with IIPs were analyzed. This cohort included 306 cases of IPF, 189 cases of unclassifiable interstitial pneumonia (UCIP), 12 cases of nonspecific interstitial pneumonia (NSIP), 13 cases of PPFE, 7 cases of cryptogenic organizing pneumonia (COP), and 1 case of desquamative interstitial pneumonia (DIP) (Figure S2). IIPs other than IPF were defined as non-IPF (222 cases). In the IPF cohort, patients were classified into low ESMI group (77 cases) and normal ESMI group (229 cases) using an ESMI value cut-off of 3.872 and into low PMI group (77 cases) and normal PMI group (229 cases) using a PMI value cut-off of 4.620. In the non-IPF cohort, patients were classified into low ESMI group (56 cases) and normal ESMI group (166 cases) using an ESMI value cut-off of 3.653 and into low PMI group (56 cases) and normal PMI group (166 cases) using a PMI value cut-off of 4.298, respectively (Fig. 1).

### Characteristics of the population

In the IPF cohort, the mean age was 72.7 years, and the proportion of males was 78.4%. The low ESMI group was significantly older, had more severe dyspnea, worse performance status (PS), and showed lower body mass index (BMI), %FEV1, %VC, %FVC, %DLCO, albumin (Alb), and hemoglobin (Hb) levels than the normal ESMI group. The low PMI group, with a higher proportion of females and no significant difference in BMI, was also older and showed lower %FEV1, %VC, %FVC, Hb, and Alb levels than the normal PMI group (Table 1, S1). In the non-IPF cohort, the mean age was 71.2 years, and the proportion of males was 67.6%. The low ESMI group was significantly older, had a higher proportion of females, and showed lower BMI, %VC, %FVC, Hb, Alb, BNP, and SP-A levels than the normal ESMI group. The low PMI group had a higher proportion of females and showed lower BMI and Hb levels than the normal PMI group (Table 2, S2).

**Table 1:** Baseline characteristics of IPF patients, according to ESMI and PMI groups

Characteristic	Overall	low ESMI	normal ESMI	*p*-value *	low PMI	normal PMI	*p*-value ^†^
N	306	77	229		77	229	
Age (years), mean ± SD	72.7 ± 7.2	74.9 ± 6.3	72.0 ± 7.3	0.002^§^	75.1 ± 6.5	71.9 ± 7.2	0.001^§^
Sex, n (%)				0.354			< 0.001^§^
Male (%)	240 (78.4)	57 (74.0)	183 (79.9)		45 (58.4)	195 (85.2)	
Female (%)	66 (21.6)	20 (26.0)	46 (20.1)		32 (41.6)	34 (14.8)	
BMI (kg/m^2^), median (IQR)	23.2 (21.1–25.2)	20.8 (18.7–23.0)	23.9 (22.2–25.7)	< 0.001^§^	23.0 (20.0-25.2)	23.2 (21.6–25.2)	0.082
Smoking pack-year, median (IQR)	40.0 (22.0–60.0)	40.0 (21.0–60.0)	40.0 (23.0–57.0)	0.798	40.0 (20.0–60.0)	40.0 (24.0–60.0)	0.500
Never, n (%)	76 (24.8)	24 (31.2)	52 (22.7)		28 (36.4)	48 (21.0)	
Former, n (%)	196 (64.1)	46 (59.7)	150 (65.5)		40 (51.9)	156 (68.1)	
Current, n (%)	34 (11.1)	7 (9.1)	27 (11.8)		9 (11.7)	25 (10.9)	
%VC (%), mean ± SD	80.7 ± 20.3	73.7 ± 21.2	83.1 ± 19.5	< 0.001^§^	78.7 ± 21.7	85.6 ± 19.8	0.011^§^
%FVC (%), mean ± SD	83.0 ± 20.6	76.1 ± 22.6	85.3 ± 19.4	0.001^§^	76.6 ± 20.7	85.0 ± 20.2	0.002^§^
%FEV1 (%), mean ± SD	83.9 ± 20.5	79.9 ± 21.7	85.3 ± 19.9	0.049^§^	74.5 ± 20.5	82.8 ± 19.9	0.002^§^
%DLCO (%), mean ± SD	62.3 ± 26.2	48.3 ± 29.8	65.4 ± 24.4	0.003^§^	56.6 ± 25.1	63.8 ± 26.4	0.205
PMI, mean ± SD	5.6 ± 1.3	5.1 ± 1.2	5.7 ± 1.3	< 0.001^§^	4.0 ± 0.6	6.1 ± 1.0	< 0.001^§^
ESMI, mean ± SD	4.6 ± 1.1	3.2 ± 0.6	5.1 ± 0.8	< 0.001^§^	4.2 ± 1.3	4.7 ± 1.1	< 0.001^§^

*IPF* idiopathic pulmonary fibrosis, *BMI* body mass index,* VC* vital capacity, *FVC* forced vital capacity, *FEV1* forced expiratory volume in 1 s, *DLCO* carbon monoxide diffusing capacity, *ESMI* erector spinae muscle index, *PMI* pectoralis muscle index

*: Comparison between low ESMI and normal ESMI groups

^†^: Comparison between low PMI and normal PMI groups

^§^: *P* < 0.05 with chi-square test, student t-test, or Man Whitney U test

Missing data is as followed, (n); %VC (2), %FVC (2), %FEV1 (2), %DLCO (175)

**Table 2:** Baseline characteristics of non-IPF patients, according to ESMI and PMI groups

Characteristic	Overall	low ESMI	normal ESMI	*p*-value *	low PMI	normal PMI	*p*-value ^†^
N	222	56	166		56	166	
Age (years), mean ± SD	71.2 ± 9.2	73.6 ± 8.9	70.4 ± 9.1	0.026^§^	72.8 ± 8.5	70.7 ± 9.3	0.154
Sex, n (%)				0.015^§^			< 0.001^§^
Male (%)	150 (67.6)	30 (53.6)	120 (72.3)		25 (44.6)	125 (75.3)	
Female (%)	72 (32.4)	26 (46.4)	46 (27.7)		31 (55.4)	41 (24.7)	
BMI (kg/㎡), median (IQR)	23.1 (20.9–25.6)	20.3 (17.7–22.3)	23.7 (21.8–26.1)	< 0.001^§^	22.2 (20.0-23.6)	23.4 (21.1–25.7)	0.006^§^
Smoking pack-year, median (IQR)	42.0 (27.2–60.0)	49.0 (26.2–71.2)	40.0 (28.0-54.4)	0.245	45.0 (33.8–59.1)	40.5 (26.2–59.6)	0.551
Never, n (%)	81 (36.5)	29 (51.8)	52 (31.3)		34 (60.7)	47 (28.3)	
Former, n (%)	118 (53.2)	25 (44.6)	93 (56.0)		20 (35.7)	98 (59.0)	
Current, n (%)	23 (10.4)	2 (3.6)	21 (12.7)		2 (3.6)	21 (12.7)	
%VC (%), mean ± SD	80.4 ± 19.6	72.6 ± 16.3	83.0 ± 19.9	< 0.001^§^	76.5 ± 17.2	81.7 ± 20.2	0.085
%FVC (%), mean ± SD	82.4 ± 20.0	74.7 ± 18.7	84.9 ± 19.4	0.001^§^	79.1 ± 18.9	83.4 ± 19.9	0.155
%FEV1 (%), mean ± SD	78.9 ± 21.2	74.6 ± 22.9	80.3 ± 20.5	0.082	80.6 ± 22.9	78.3 ± 20.7	0.488
%DLCO (%), mean ± SD	69.0 ± 22.1	64.2 ± 17.8	70.5 ± 23.2	0.293	66.2 ± 19.3	69.7 ± 22.9	0.575
PMI, mean ± SD	5.3 ± 1.3	4.5 ± 1.0	5.5 ± 1.3	< 0.001^§^	3.7 ± 0.4	5.8 ± 1.1	< 0.001^§^
ESMI, mean ± SD	4.5 ± 1.2	3.0 ± 0.5	5.1 ± 0.9	< 0.001^§^	3.8 ± 1.1	4.8 ± 1.2	< 0.001^§^

*IPF* idiopathic pulmonary fibrosis, *ESMI* erector spinae muscle index, *PMI* pectoralis muscle index, *BMI* body mass index, *VC* vital capacity, *FVC* forced vital capacity, *FEV1* forced expiratory volume in 1 s, *DLCO* carbon monoxide diffusing capacity

*: Comparison between low ESMI and normal ESMI groups

^†^: Comparison between low PMI and normal PMI groups

^§^:*P* < 0.05 with chi-square test, student t-test, or Man Whitney U test

Missing date is as followed, (n); pack-year (82), %VC (1), %FVC (1), %FEV1 (1), %DLCO (144)

### Association of ESMI and PMI with 5-year survival and acute exacerbation

**Fig. 2:**
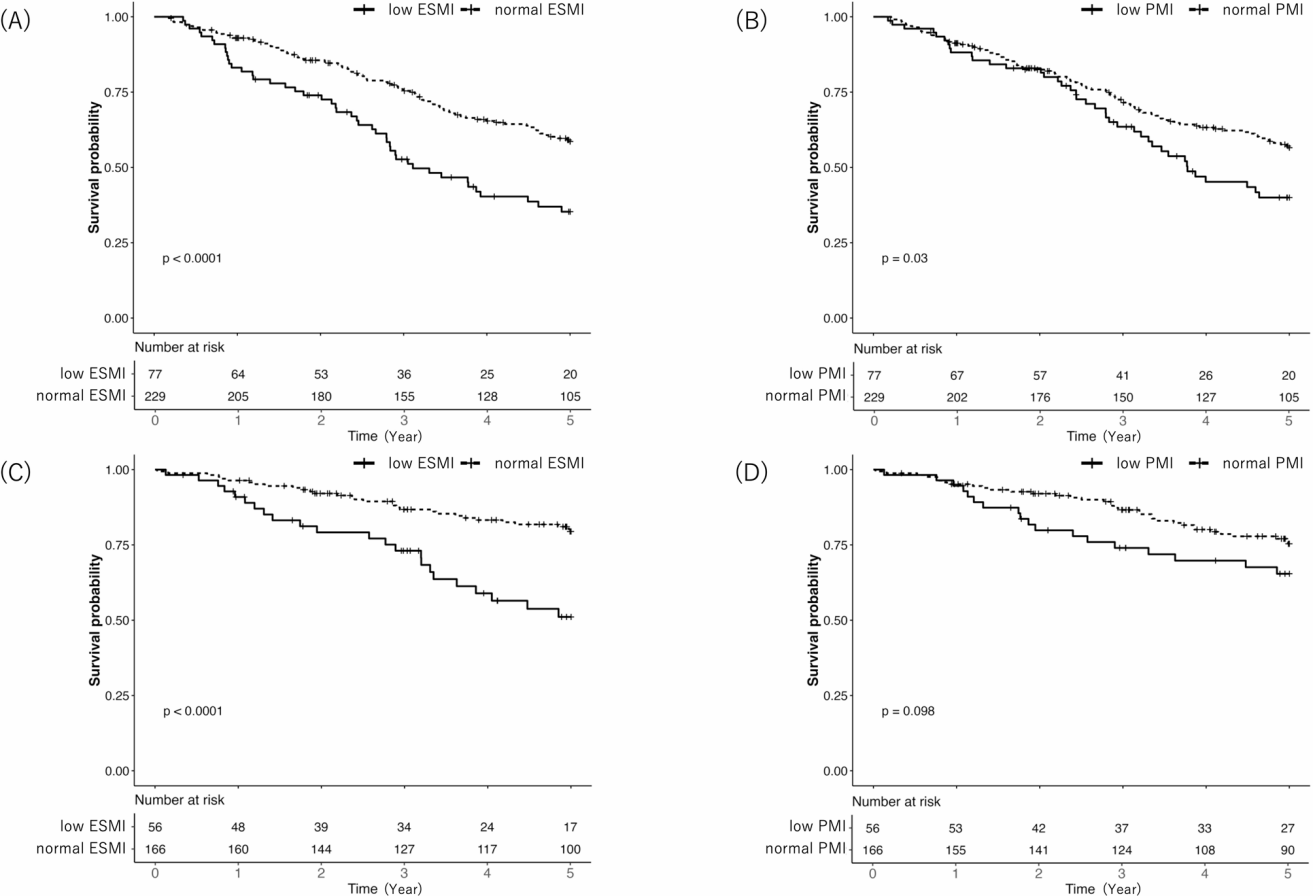
Kaplan–Meier plots of survival probability according to baseline lung function categories Kaplan- Meier curves of survival probability according to ESMI and PMI in patients with IPF (**A**, **B**) and non-IPF (**C**, **D**) ESMI, erector spinae muscle index; PMI, pectoralis muscle index; IPF, idiopathic pulmonary fibrosis

Of the 306 patients with IPF, 125 had completed the 5-year follow-up, and 132 had died over 1,070.7 person-years, whereas 117 out of the 222 non-IPF IIP patients had completed the 5-year follow-up, and 54 had died over 870.1 person-years. In the IPF cohort, the 5-year survival rate was 58.6% (95% CI, 52.2–65.8%) in the normal ESMI group, 35.3% (95% CI, 25.5–48.8%) in the low ESMI group, 56.6% (95% CI, 50.2–63.7%) in the normal PMI group, and 40.0% (95% CI, 29.5–54.2%) in the low PMI group (Fig. 2A and B). In the non-IPF cohort, the 5-year survival rate was 79.5% (95% CI, 73.2–86.2%) in the normal ESMI group, 51.1% (95% CI, 38.2–68.2%) in the low ESMI group, 75.4% (95% CI, 68.6–82.8%) in the normal PMI group, and 65.4% (95% CI, 53.6–79.9%) in the low PMI group (Fig. 2C and D). There were seven patients with BMI ≥ 25 combined with low ESMI in both the IPF and non-IPF cohort, and BMI ≥ 25 combined with low PMI was observed in 23 patients in the IPF cohort and 10 patients in the non-IPF cohort.

The HR for mortality in the normal ESMI group, adjusted for age, sex, smoking level, and %FVC, was 0.62 (95%CI, 0.40–0.90) in the IPF cohort and 0.46 (95%CI, 0.26–0.83) in the non-IPF cohort, considering the low ESMI groups as references. In contrast, the adjusted HR for mortality in the normal PMI group was 0.86 (95%CI, 0.58–1.28) in the IPF cohort and 0.67 (95%CI, 0.37–1.22) in the non-IPF cohort, showing no statistically significant difference in either cohort (Table 3). Restricted cubic spline analyses detected no significant non-linearity for either ESMI (IPF: χ² = 0.73, *p* = 0.70; non-IPF: χ² = 0.26, *p* = 0.88) or PMI (IPF: χ² = 2.33, *p* = 0.31; non-IPF: χ² = 0.49, *p* = 0.78); consequently, no clear thresholds were identified for either variable. In the IPF cohort, an adjusted Cox proportional-hazards model treating ESMI and PMI as continuous variables demonstrated that PMI, in addition to ESMI, was a predictor of overall survival (Table S3).

**Table 3:** Hazard ratios and 95% confidence intervals for the mortality of the normal muscle mass groups compared to the low muscle mass groups in the IPF and Non-IPF cohort

IIPs type	Covariates	*N*	ESMI	PMI
			HR (95% CI)	HR (95% CI)
IPF	None	306	0.50 (0.35–0.71)	0.66 (0.46–0.96)
	Sex, Age, %FVC, Smoking level	304	0.62 (0.40–0.90)	0.86 (0.58–1.28)
non-IPF	None	222	0.35 (0.20–0.60)	0.62 (0.35–1.10)
	Sex, Age, %FVC, Smoking level	221	0.46 (0.26–0.83)	0.67 (0.37–1.22)

*IPF* idiopathic pulmonary fibrosis,* IIPs* idiopathic interstitial pneumonias, *ESMI* erector spinae muscle index, *PMI* pectoralis muscle index, *HR* hazard ratio, *CI* confidence interval, *FVC* forced vital capacity

The predictors of mortality in the IPF and non-IPF cohorts were examined using multivariate Cox regression analysis (stepwise variable selection). In the IPF cohort, ESMI, Hb, Alb, LDH, %FEV1, and %FVC were independently associated with mortality. In the non-IPF cohort, ESMI, age, comorbid heart disease, C-reactive protein, KL-6, and %FVC were independently associated with mortality (Table 4). Regarding the causes of death, there was an increase in mortality related to the underlying IIPs, such as chronic respiratory failure, acute exacerbations, and lung cancer, as well as in causes not directly associated with the underlying disease in the low ESMI group compared to the normal ESMI group in both cohorts. Across all disease cohorts and ESMI categories, chronic respiratory failure was the most common cause of death, followed by acute exacerbations (Table S4).

**Table 4:** Cox regression analysis for mortality of the IPF cohort and the non-IPF cohort according to baseline characteristics after Stepwise selection

Characteristics	Hazard ratio (95%CI)	*p*-value
IPF (*n* = 294)		
ESMI	0.77 (0.65–0.92)	0.004
Hb	1.24 (1.09–1.41)	0.002
Alb	0.40 (0.24–0.64)	< 0.001
LDH	1.01 (1.00-1.01)	< 0.001
%FEV1	1.02 (1.01–1.05)	0.015
%FVC	0.94 (0.92–0.96)	< 0.001
Non-IPF (*n* = 221)		
ESMI	0.65 (0.50–0.85)	0.002
Age	1.08 (1.05–1.12)	< 0.001
Heart disease	2.17 (1.15–4.09)	0.016
CRP	1.29 (1.11–1.52)	< 0.001
KL-6	1.0001 (1.000-1.001)	0.002
%FVC	0.97 (0.95–0.99)	< 0.001

*IPF* idiopathic pulmonary fibrosis, *CI* confidence interval, *ESMI* erector spinae muscle index, *Hb* haemoglobin, *Alb* albumin, *LDH* lactate dehydrogenase, *FEV1* forced expiratory volume 1 s, *FVC* forced vital capacity, *CRP* C-reactive protein

In the IPF cohort, the cumulative incidence rate of acute exacerbations at 5 years was 30.4% (95% CI, 22.5–37.4%) in the normal ESMI group, 37.3% (95% CI, 21.8–49.7%) in the low ESMI group, 29.7% (95% CI, 22.1–36.6%) in the normal PMI group, and 42.4% (95% CI, 24.1–56.3%) in the low PMI group. In the non-IPF cohort, the cumulative incidence rate of acute exacerbations at 5 years was 19.7% (95% CI, 12.5–26.4%) in the normal ESMI group, 48.6% (95% CI, 26.2–64.2%) in the low ESMI group, 21.3% (95% CI, 13.6–28.3%) in the normal PMI group, and 38.6% (95% CI, 20.7–52.5%) in the low PMI group (Figure S3). After adjustment for age, sex, %FVC, and smoking level, the HRs for acute exacerbation were 0.43 (95% CI, 0.22–0.83) in the normal ESMI group and 0.51 (95% CI, 0.26–0.99) in the low PMI group, considering the low ESMI and low PMI groups as the reference in the non-IPF cohort (Table S5).

### Association between ESMI, PMI, and clinical features

In both the IPF and non-IPF cohorts, ESMI and baseline %FVC showed a weak correlation (*r* = 0.187, 0.198, respectively). PMI demonstrated a weak correlation with %FVC only in the IPF cohort (*r* = 0.123), and the correlation between PMI and %FVC was weaker than that of ESMI and %FVC (Figure S4). In both the IPF and non-IPF cohorts, a higher ESMI was significantly associated with a lower mMRC score after adjusting for age, sex, and %FVC (HR: 0.71, 95%CI, 0.57–0.90; HR: 0.73, 95%CI, 0.54–0.97, respectively). In contrast, the correlation between PMI and mMRC was not statistically significant in either cohort (Table S6).

## Discussion

This multicenter study enrolled patients centrally diagnosed through MDD and prospectively followed 306 IPF cases and 222 non-IPF cases over 5 years for survival and exacerbations. Not only in the IPF cohort, but also in the non-IPF cohort the normal ESMI group demonstrated significantly longer survival compared to the low ESMI group after adjusting for age, sex, smoking level, and %FVC. In contrast, no significant difference in survival was observed between the normal PMI and low PMI groups in either cohort. Multivariate Cox regression analysis further confirmed that ESMI was an independent predictor of survival in both IPF and non-IPF cohorts. This study also revealed that ESMI and PMI also serves as a predictive factor for acute exacerbations, particularly in non-IPF patients.

Several studies similar to the present investigation have previously been conducted in patients with IPF [16–18]. However, as far as we are aware, studies exploring the association between skeletal muscle mass and prognosis in non-IPF IIPs are limited to a single report on iPPFE [11]. While skeletal muscle mass at the level of the third lumbar vertebra (L3) is considered a reliable indicator of sarcopenia [26, 27], many studies on skeletal muscle mass in chronic respiratory diseases utilize chest CT to avoid additional radiation exposure. Although there is no established consensus regarding which region should be measured for chest skeletal muscle mass quantification, several reports have evaluated muscle mass at the T12 level, which is closest to L3 in the chest CT imaging range, as well as at the T4 level [16–19, 28]. In previous studies, Moon et al. demonstrated that skeletal muscle mass at the T4 level was associated with poor prognosis in Korean patients with IPF, while low muscle mass at the T12 level was not statistically associated with poor prognosis [16]. Similarly, Fujikawa et al. reported that the cross-sectional area of the pectoralis muscle is associated with the prognosis of patients with IPF [20]. In contrast, Awano et al. reported that low erector spinae muscle was significantly associated with poor prognosis, whereas low pectoralis muscle did not show a statistically significant correlation with prognosis [29]. In this study, we used the height-adjusted cross-sectional area of the pectoralis muscle at the T4 level and the erector spinae muscle at the T12 level, expressed as ESMI and PMI, as indicators. As a result, we demonstrated that ESMI is an independent prognostic factor, whereas PMI was not. When muscle-mass indices were analysed as continuous variables, PMI remained an independent predictor of mortality only in the IPF cohort, presumably owing to the greater statistical power obtained by avoiding categorisation. In multivariable-adjusted stepwise Cox models, ESMI retained its prognostic significance independent of %FVC and other covariates, whereas PMI was not selected in either the IPF or non-IPF cohort. Taken together, these findings provide robust evidence that ESMI is a prognostic marker in our cohort. These results may be attributed to ESMI being a trunk muscle, which reflects total body muscle mass more accurately than PMI. In this study, we evaluated the prognosis of the non-IPF group as a single entity, with 85.1% of cases comprising UCIP, a condition known to exhibit diverse clinical courses [30–32]. This study not only validates the previously reported prognostic significance of ESMI in IPF but also demonstrates its role as a poor prognostic factor in the non-IPF cohort. These findings support the potential utility of ESMI as a critical prognostic indicator across the spectrum of fibrosing interstitial pneumonia. Although not addressed in the present study, determining whether ESMI or appendicular skeletal muscle mass index, a commonly used indicator of muscle mass in sarcopenia, more accurately reflects prognosis remains an important issue for future research to establish the clinical utility of ESMI. Moreover, we revealed that ESMI and PMI also serves as a predictive factor for acute exacerbations, particularly in non-IPF. To the best of our knowledge, this is the first study to elucidate the relationship between acute exacerbations and muscle mass. Further studies are warranted to validate whether muscle mass can function as a reliable predictor of exacerbations.

ESMI demonstrated a weak positive correlation with %FVC in both the IPF and non-IPF cohorts, which was more pronounced than the correlation between PMI and %FVC. ESMI was also found to negatively correlate with mMRC score in both groups, whereas PMI did not show a statistically significant correlation with mMRC score in either group. Since ESMI reflects the musculature of the trunk, it may be more closely related to exercise tolerance and physical activity than PMI. To the best of our knowledge, no prior studies have directly demonstrated an association between ESMI and exercise tolerance or physical activity. Dyspnea has been reported to impact health-related quality of life (HRQoL) and physical activity [33, 34]. Although low ESMI was associated with greater dyspnea in our cohort, the present data do not demonstrate correlation of ESMI of HRQoL or physical activity; further research is needed to clarify these relationships. Although therapeutic interventions such as pulmonary rehabilitation might help preserve skeletal muscle mass and functional capacity, their impact was not assessed in this study and should be explored in future research.

This study has several limitations. First, data on exercise tolerance, grip stremgth, physical activity, HRQoL, and rehabilitation interventions, which precluded an evaluation of the relationship between ESMI and these clinical indicators. Second, standardized reference ranges for ESMI have not yet been established, restricting the generalizability of our findings. Third, because the time of diagnosis was not recorded, we could not adjust for disease duration from diagnosis, raising the possibility of length-bias survival effects. Fourth, the study included only patients who provided informed consent rather than consecutive patients, potentially limiting the generalisability of our conclusions. Finally, despite the protocolized assessments, some data were missing mostly likely at random.

### Conclusions

We demonstrate that ESMI is an independent factor associated with survival prognosis in both IPF patients and non-IPF patients. Lower ESMI and PMI were additionally associated with acute exacerbations in non-IPF patients. Furthermore, ESMI correlates with exercise-induced dyspnea and pulmonary function. These findings highlight the significance of ESMI as a simple and practical prognostic factor for both survival and exacerbations in patients with IIPs, emphasizing the need for proactive interventions in those with low trunk muscle mass.

## Supplementary Information

Supplementary Material 1. Table S1. Baseline characteristics of IPF patients, according to ESMI and PMI groups.

Supplementary Material 2. Table S2. Baseline characteristics of non-IPF patients, according to ESMI and PMI groups.

Supplementary Material 3. Table S3. Hazard ratios (95 % CI) for mortality with ESMI and PMI modeled as continuous variables in IPF and non-IPF cohorts

Supplementary Material 4. Table S4. Cause of death

Supplementary Material 5. Table S5. Hazard ratios and 95% confidence intervals for the acute exacerbation of the normal muscle mass groups compared to the low muscle mass groups in the IPF and non-IPF cohort.

Supplementary Material 6. Odds Ratios and their 95% confidence Intervals for ESMI and PMI in relation to mMRC score using an ordinal logistic regression model for the low muscle mass groups compared to the normal muscle mass groups in the IPF and non-IPF cohort.

Supplementary Material 7. Figure S1. Sample images of CT scans used for measurement of muscle areas pectoralis muscles cross section areas at T4 vertebra (A) and paraspinal muscles at T12 vertebra (B).

Supplementary Material 8. Figure S2. Diagnosis for the study patients after multidisciplinary discussion. IPF, idiopathic pulmonary fibrosis; IIPs, idiopathic interstitial pneumonias. PPFE, pleuroparenchymal fibroelastosis; NSIP, nonspecific interstitial pneumonia; COP, cryptogenic organising pneumonia; DIP, desquamative interstitial pneumonia.

Supplementary Material 9. Figure S3. Kaplan- Meier plot for the cumulative incidence of acute exacerbation stratified by ESMI or PMI groups.Kaplan- Meier curves plot for the cumulative incidence of acute exacerbation according to ESMI and PMI in patients with IPF (A, B) and non-IPF (C, D). IPF, idiopathic pulmonary fibrosis; ESMI, erector spinae muscle index; PMI, pectoralis muscle index. IPF, idiopathic pulmonary fibrosis.

Supplementary Material 10. Figure S4. Correlation between %FVC and ESMI or PMI according to IPF and non-IPF cohort. %FVC, forced vital capacity; ESMI, erector spinae muscle index; PMI, pectoralis muscle index.

## Data Availability

The datasets used and/or analyzed during the current study are available from the corresponding author on reasonable request.

## Abbreviations

IPF: Idiopathic pulmonary fibrosis
IIPs: Idiopathic interstitial pneumonias
ILDs: Interstitial lung diseases
CT: Computed tomography
ESMI: Erector spinae muscle index
PMI: Pectoralis muscle index
FVC: Forced vital capacity
HR: Hazard ratio
iPPFE: Idiopathic pleuroparenchymal fibroelastosis
MDD: Multidisciplinary discussion
COPD: Chronic obstructive pulmonary disease
MDD: Multidisciplinary discussion
FEV1: Forced expiratory volume 1 second
VC: Vital capacity
JRS: Japanese Respiratory Society
BNP: Brain natriuretic peptide
SP-D: Surfactant protein-D
SP-A: Surfactant protein-A
mMRC: modified Medical Research Council score
UCIP: Unclassifiable interstitial pneumonia
NSIP: Nonspecific interstitial pneumonia
COP: Cryptogenic organizing pneumonia
DIP: Desquamative interstitial pneumonia
PS: Performance status
BMI: Body mass index
Alb: Albumin
Hb: Hemoglobin
