# Supplementary material for: Prognostic impact of muscle mass in idiopathic interstitial pneumonia: analysis of idiopathic pulmonary fibrosis and other idiopathic interstitial pneumonias
Source: BMC Pulm Med. 2025 Oct 14;25:468. doi: 10.1186/s12890-025-03942-0 (PMC12522827; doi:10.1186/s12890-025-03942-0)
Supplement: Supplementary file 1 — Supplementary Material 1. Table S1. Baseline characteristics of IPF patients, according to ESMI and PMI groups. [file 12890_2025_3942_MOESM1_ESM.docx]

**Table S1. Baseline characteristics of IPF patients, according to ESMI and PMI groups.**

| Characteristic | Overall | low  ESMI | normal  ESMI | p-  value  * | low  PMI | normal  PMI | p-  value  ^†^ |
| --- | --- | --- | --- | --- | --- | --- | --- |
| N | 306 | 77 | 229 |  | 77 | 229 |  |
| mMRC  , n (%) |  |  |  | <0.001^§^ |  |  | 0.396 |
| 0 | 39 (17.9) | 4  (6.8) | 35 (22.0) |  | 10  (17.5) | 29 (18.0) |  |
| 1 | 88 (40.4) | 18 (30.5) | 70 (44.0) |  | 21 (36.8) | 67 (41.6) |  |
| 2 | 55 (25.2) | 17 (28.8) | 38 (23.9) |  | 13 (22.8) | 42 (26.1) |  |
| 3 | 24 (11.0) | 11  (18.6) | 13  (8.2) |  | 7  (12.3) | 17 (10.6) |  |
| 4 | 12  (5.5) | 9  (15.3) | 3  (1.9) |  | 6  (10.5) | 6  (3.7) |  |
| PS, n (%) |  |  |  | 0.025^§^ |  |  | 0.070 |
| 0 | 144 (47.1) | 31 (40.3) | 113 (49.3) |  | 30 (39.0) | 114 (49.8) |  |
| 1 | 136 (44.4) | 32  (41.6) | 104 (45.4) |  | 38  (49.4) | 98 (42.8) |  |
| 2 | 19  (6.2) | 9  (11.7) | 10  (4.4) |  | 5  (6.5) | 14  (6.1) |  |
| 3 | 7  (2.3) | 5  (6.5) | 2  (0.9) |  | 4  (5.2) | 3  (1.3) |  |
| 4 | 0  (0.0) | 0  (0.0) | 0  (0.0) |  | 0  (0.0) | 0  (0.0) |  |
| Comorbidities, n (%) |  |  |  |  |  |  |  |
| Diabetes | 45 (14.7) | 8  (10.4) | 37  (16.2) | 0.294 | 16  (20.8) | 29  (12.7) | 0.120 |
| Dislipidemia | 57 (18.6) | 13  (16.9) | 44  (19.2) | 0.775 | 19  (24.7) | 38  (16.6) | 0.160 |
| Hypertension | 99 (32.4) | 27  (35.1) | 72  (31.4) | 0.655 | 25  (32.5) | 74  (32.3) | 1.000 |
| Heart disease | 47 (15.4) | 15  (19.5) | 32  (14.0) | 0.329 | 11  (14.3) | 36  (15.7) | 0.905 |
| Reflux esophagitis | 20  (6.5) | 5  (6.5) | 15  (6.6) | 1.000 | 5  (6.5) | 15  (6.6) | 1.000 |
| Hb (g/dl),  mean±SD | 13.8  ±1.6 | 13.3  ±1.7 | 14.0  ±1.5 | 0.002^§^ | 13.3  ±1.5 | 14.0  ±1.5 | 0.001^§^ |
| Alb (g/dl),  mean±SD | 4.0  ±0.4 | 3.9  ±0.4 | 4.0  ±0.4 | 0.003^§^ | 3.9  ±0.4 | 4.0  ±0.4 | 0.018^§^ |
| LDH (U/l),  median  (IQR) | 211.0  (187.8-239.0) | 200.5  (178.8-239.5) | 213.0  (191.0-239.0) | 0.058 | 212.5  (180.8-254.5) | 210.0  (189.0-233.2) | 0.825 |
| CRP (mg/dl),  median (IQR) | 0.2 (0.1-0.3) | 0.2 (0.1-0.3) | 0.1 (0.1-0.3) | 0.465 | 0.2 (0.1-0.4) | 0.1 (0.1-0.3) | 0.222 |
| BNP (pg/ml), median  (IQR) | 22.3 (13.3-39.6) | 28.1 (17.0-41.0) | 20.0 (13.1-39.2) | 0.327 | 27.2 (16.2-42.8) | 19.8 (13.2-36.4) | 0.298 |
| NT-proBNP (pg/ml), median  (IQR) | 90.0 (51.0-163.0) | 97.0 (59.0-178.0) | 81.5 (49.8-152.8) | 0.475 | 115.1 (64.0-162.2) | 81.0 (48.5-162.0) | 0.118 |
| KL-6 (U/ml) ,  median (IQR) | 759.0 (501.0-1161.0) | 678.0 (461.0-1095.5) | 803.0 (510.8-1174.2) | 0.149 | 737.0 (473.2-1124.8) | 766.0 (511.5-1161.0) | 0.479 |
| SP-A (ng/ml) ,  median (IQR) | 60.6 (46.6-84.8) | 56.1 (51.1-71.4) | 62.6 (45.6-88.8) | 0.515 | 61.3 (46.4-97.4) | 59.8 (47.2-84.5) | 0.976 |
| SP-D (ng/ml) ,  median (IQR) | 186.0 (118.8-323.0) | 141.0 (95.5-295.8) | 192.0 (120.5-324.0) | 0.211 | 142.1 (122.0-232.6) | 194.0 (118.4-338.5) | 0.141 |

IPF, idiopathic pulmonary fibrosis; ESMI, erector spinae muscle index, PMI, pectoralis muscle index; mMRC, modified medical research council; PS, performance status; Hb, haemoglobin; Alb, albumin; LDH, lactate dehydrogenase; CRP, C-reactive protein; BNP, brain natriuretic peptide; NT-pro BNP, N terminal-pro BNP; SP-A, Surfactant Protein-A; SP-D, Surfactant Protein-D.

*: Comparison between low ESMI and normal ESMI groups

^†^: Comparison between low PMI and normal PMI groups

§: P<0.05 with chi-square test, student t-test

Missing date is as followed, (n); mMRC (88), Hb (2), Alb (5), LDH (2), CRP (2), BNP (140), NT-proBNP (169), KL-6 (3), SP-A (220), SP-D (185).
