# Supplementary material for: Prognostic impact of muscle mass in idiopathic interstitial pneumonia: analysis of idiopathic pulmonary fibrosis and other idiopathic interstitial pneumonias
Source: BMC Pulm Med. 2025 Oct 14;25:468. doi: 10.1186/s12890-025-03942-0 (PMC12522827; doi:10.1186/s12890-025-03942-0)
Supplement: Supplementary file 2 — Supplementary Material 2. Table S2. Baseline characteristics of non-IPF patients, according to ESMI and PMI groups. [file 12890_2025_3942_MOESM2_ESM.docx]

**Table S2. Baseline characteristics of non-IPF patients, according to ESMI and PMI groups.**

| Characteristic | Overall | low  ESMI | normal  ESMI | p-  value  ^§^ | low  PMI | normal  PMI | p-  value  ^§^ |
| --- | --- | --- | --- | --- | --- | --- | --- |
| N | 222 | 56 | 166 |  | 56 | 166 |  |
| mMRC  , n (%) |  |  |  | 0.058 |  |  | 0.223 |
| 0 | 23 (14.6) | 1  (2.5) | 22 (18.8) |  | 4  (9.3) | 19 (16.7) |  |
| 1 | 83 (52.9) | 21 (52.5) | 62  (53.0) |  | 22 (51.2) | 61 (53.5) |  |
| 2 | 27 (17.2) | 8  (20.0) | 19  (16.2) |  | 6  (14.0) | 21 (18.4) |  |
| 3 | 20  (12.7) | 8  (20.0) | 12  (10.3) |  | 9  (20.9) | 11  (9.6) |  |
| 4 | 4  (2.5) | 2  (5.0) | 2  (1.7) |  | 2  (4.7) | 2  (1.8) |  |
| PS, n (%) |  |  |  | 0.094 |  |  | 0.123 |
| 0 | 98 (44.1) | 21 (37.5) | 77  (46.4) |  | 21 (37.5) | 77 (46.4) |  |
| 1 | 104 (46.8) | 26 (46.4) | 78 (47.0) |  | 27 (48.2) | 77 (46.4) |  |
| 2 | 15 (6.8) | 6 (10.7) | 9  (5.4) |  | 4  (7.1) | 11 (6.6) |  |
| 3 | 4  (1.8) | 3  (5.4) | 1  (0.6) |  | 4  (7.1) | 0  (0.0) |  |
| 4 | 1  (0.5) | 0  (0.0) | 1  (0.6) |  | 0  (0.0) | 1  (0.6) |  |
| Comorbidities, n (%) |  |  |  |  |  |  |  |
| Diabetes | 32  (14.4) | 7  (12.5) | 25  (15.1) | 0.801 | 7  (12.5) | 25  (15.1) | 0.801 |
| Dislipidemia | 52  (23.4) | 12  (21.4) | 40  (24.1) | 0.822 | 14  (25.0) | 38  (22.9) | 0.889 |
| Hypertension | 79  (35.6) | 23  (41.1) | 56  (33.7) | 0.406 | 24  (42.9) | 55  (33.1) | 0.249 |
| Heart disease | 34  (15.3) | 7  (12.5) | 27  (16.3) | 0.644 | 8  (14.3) | 26  (15.7) | 0.974 |
| Reflux esophagitis | 20  (9.0) | 8  (14.3) | 12  (7.2) | 0.185 | 7  (12.5) | 13  (7.8) | 0.432 |
| Hb (g/dl),  mean±SD | 13.8  ±1.6 | 13.1  ±1.6 | 14.1  ±1.5 | <0.001^§^ | 13.3  ±1.6 | 14.0  ±1.6 | 0.008^§^ |
| Alb (g/dl) ,  mean±SD | 4.1  ±0.4 | 3.9  ±0.4 | 4.1  ±0.4 | 0.005^§^ | 4.1  ±0.4 | 4.1  ±0.4 | 0.886 |
| LDH (U/l),  median  (IQR) | 208.5  (188.3-236.0) | 204.5  (180.8-232.0) | 212.0  (191.2-238.0) | 0.109 | 205.0  (181.0-236.0) | 210.0  (191.2-236.5) | 0.417 |
| CRP (mg/dl),  median (IQR) | 0.1 (0.1-0.3) | 0.1 (0.1-0.3) | 0.1 (0.1-0.3) | 0.579 | 0.1 (0.1-0.3) | 0.1 (0.1-0.3) | 0.735 |
| BNP (pg/ml), median  (IQR) | 20.0 (12.2-34.6) | 31.2 (19.8-49.5) | 17.0 (11.7-28.8) | 0.001^§^ | 23.5 (16.0-46.4) | 17.5 (12.0-29.3) | 0.068 |
| NT-proBNP (pg/ml), median  (IQR) | 85.0 (50.5-134.0) | 100.0 (68.0-163.0) | 76.0 (48.0-123.0) | 0.103 | 82.5 (54.0-128.2) | 85.0 (48.4-137.9) | 0.929 |
| KL-6 (U/ml) ,  median (IQR) | 695.5 (417.0-1126.8) | 611.0 (346.5-1080.2) | 725.5 (455.2-1130.5) | 0.150 | 602.5 (365.0-930.2) | 739.0 (438.2-1236.2) | 0.110 |
| SP-A (ng/ml) ,  median (IQR) | 52.8 (39.5-84.8) | 39.0 (30.3-47.2) | 61.7 (41.5-85.0) | 0.020^§^ | 56.2 (36.3-81.3) | 52.8 (40.4-84.8) | 0.982 |
| SP-D (ng/ml) ,  median (IQR) | 179.5 (116.3-276.5) | 172.0 (120.0-277.0) | 181.0 (114.0-275.0) | 0.974 | 152.0 (87.4  -210.0) | 213.0 (127.0-296.8) | 0.074 |

IPF, idiopathic pulmonary fibrosis; ESMI, erector spinae muscle index, PMI, pectoralis muscle index; mMRC, modified medical research council; PS, performance status; Hb, haemoglobin; Alb, albumin; LDH, lactate dehydrogenase; CRP, C-reactive protein; BNP, brain natriuretic peptide; NT-pro BNP, N terminal-pro BNP; SP-A, Surfactant Protein-A; SP-D, Surfactant Protein-D.

*: Comparison between low ESMI and normal ESMI groups

^†^: Comparison between low PMI and normal PMI groups

^§^: P<0.05 with chi-square test, student t-test, or Man Whitney U test

NA, not available

Missing date is as followed, (n); mMRC (65), BNP (95), NT-proBNP (130), SP-A (157), SP-D (146).
