# Supplementary material for: Prognostic impact of muscle mass in idiopathic interstitial pneumonia: analysis of idiopathic pulmonary fibrosis and other idiopathic interstitial pneumonias
Source: BMC Pulm Med. 2025 Oct 14;25:468. doi: 10.1186/s12890-025-03942-0 (PMC12522827; doi:10.1186/s12890-025-03942-0)
Supplement: Supplementary file 3 — Supplementary Material 3. Table S3. Hazard ratios (95 % CI) for mortality with ESMI and PMI modeled as continuous variables in IPF and non-IPF cohorts [file 12890_2025_3942_MOESM3_ESM.docx]

**Table S3.** **Hazard ratios (95 % CI) for mortality with ESMI and PMI modeled as continuous variables in IPF and non-IPF cohorts**

| IIPs type | Covariates | N | ESMI | PMI |
| --- | --- | --- | --- | --- |
|  |  |  | HR (95% CI) | HR (95% CI) |
| IPF | Sex, Age, %FVC, Smoking level | 304 | 0.75  (0.65-0.88) | 0.85  (0.73-0.98) |
| non-IPF | Sex, Age, %FVC, Smoking level | 221 | 0.67  (0.52-0.86) | 0.88  (0.69-1.10) |

IPF, idiopathic pulmonary fibrosis; IIPs, idiopathic interstitial pneumonias; ESMI, erector spinae muscle index, PMI, pectoralis muscle index; HR, hazard ratio; CI, confidence interval; FVC, forced vital capacity.
