# Supplementary material for: Prognostic impact of muscle mass in idiopathic interstitial pneumonia: analysis of idiopathic pulmonary fibrosis and other idiopathic interstitial pneumonias
Source: BMC Pulm Med. 2025 Oct 14;25:468. doi: 10.1186/s12890-025-03942-0 (PMC12522827; doi:10.1186/s12890-025-03942-0)
Supplement: Supplementary file 4 — Supplementary Material 4. Table S4. Cause of death [file 12890_2025_3942_MOESM4_ESM.docx]

**Table S4. Cause of death**

|  | IPF | | | Non-IPF | | |
| --- | --- | --- | --- | --- | --- | --- |
| Cause of death, n (%) | all | low  ESMI | normal  ESMI | all | low  ESMI | normal  ESMI |
| All cause of death | 132  (43.1) | 46  (60.5) | 86  (37.9) | 54  (24.3) | 23  (41.1) | 31  (18.7) |
| Chronic respiratory failure | 50  (37.9) | 17  (37.0) | 33  (38.4) | 16  (29.6) | 6  (26.1) | 10  (32.3) |
| Acute exacerbation | 25  (18.9) | 8  (17.4) | 17  (19.8) | 7  (13.0) | 3  (13.0) | 4  (12.9) |
| Lung cancer | 8  (6.1) | 3  (6.5) | 5  (5.8) | 1  (1.9) | 1  (4.3) | 0  (0.0) |
| Bacterial pneumonia | 12  (9.1) | 6  (13.0) | 6  (7.0) | 6  (11.1) | 2  (8.7) | 4  (12.9) |
| Pulmonary hypertension | 4  (3.0) | 0  (0.0) | 4  (4.7) | 2  (3.7) | 1  (4.3) | 1  (3.2) |
| Chronic heart failure | 3  (2.3) | 1  (2.2) | 4  (2.3) | 2  (3.7) | 2  (8.7) | 0  (0.0) |
| Unknown | 12  (9.1) | 3  (6.5) | 9  (10.5) | 8  (14.8) | 3  (13.0) | 5  (16.1) |
| Other | 18  (13.6) | 8  (17.4) | 10  (11.6) | 12  (22.2) | 5  (21.7) | 7  (22.6) |

IPF, idiopathic pulmonary fibrosis; ESMI, erector spinae muscle index, PMI, pectoralis muscle index.
