# Supplementary material for: Prognostic impact of muscle mass in idiopathic interstitial pneumonia: analysis of idiopathic pulmonary fibrosis and other idiopathic interstitial pneumonias
Source: BMC Pulm Med. 2025 Oct 14;25:468. doi: 10.1186/s12890-025-03942-0 (PMC12522827; doi:10.1186/s12890-025-03942-0)
Supplement: Supplementary file 5 — Supplementary Material 5. Table S5. Hazard ratios and 95% confidence intervals for the acute exacerbation of the normal muscle mass groups compared to the low muscle mass groups in the IPF and non-IPF cohort. [file 12890_2025_3942_MOESM5_ESM.docx]

**Table S5. Hazard ratios and 95% confidence intervals for the acute exacerbation of the normal muscle mass groups compared to the low muscle mass groups in the IPF and non-IPF cohort.**

| IIPs type | Covariates | N | ESMI | PMI |
| --- | --- | --- | --- | --- |
|  |  |  | HR (95% CI) | HR (95% CI) |
| IPF | None | 306 | 0.61  (0.36-1.03) | 0.69  (0.40-1.17) |
|  | Sex, Age, %FVC, Smoking level | 304 | 0.79  (0.46-1.38) | 0.92  (0.75-1.12) |
| non-IPF | None | 222 | 0.35  (0.20-0.60) | 0.50  (0.27-0.94) |
|  | Sex, Age, %FVC, Smoking level | 221 | 0.43  (0.22-0.83) | 0.51 (0.26-0.99) |

IPF, idiopathic pulmonary fibrosis; IIPs, idiopathic interstitial pneumonias; ESMI, erector spinae muscle index, PMI, pectoralis muscle index; HR, hazard ratio; CI, confidence interval; FVC, forced vital capacity; LDH, lactate dehydrogenase.
