# Supplementary material for: Prognostic impact of muscle mass in idiopathic interstitial pneumonia: analysis of idiopathic pulmonary fibrosis and other idiopathic interstitial pneumonias
Source: BMC Pulm Med. 2025 Oct 14;25:468. doi: 10.1186/s12890-025-03942-0 (PMC12522827; doi:10.1186/s12890-025-03942-0)
Supplement: Supplementary file 6 — Supplementary Material 6. Odds Ratios and their 95% confidence Intervals for ESMI and PMI in relation to mMRC score using an ordinal logistic regression model for the low muscle mass groups compared to the normal muscle mass groups in the IPF and non-IPF cohort. [file 12890_2025_3942_MOESM6_ESM.docx]

**Table S6. Odds Ratios and their 95% confidence Intervals for ESMI and PMI in relation to mMRC score using an ordinal logistic regression model for the low muscle mass groups compared to the normal muscle mass groups in the IPF and non-IPF cohort.**

| IIPs type | Covariates | n | ESMI  OR (95% CI) | PMI  OR (95% CI) |
| --- | --- | --- | --- | --- |
| IPF | None | 218 | 0.66  (0.53-0.82) | 0.81  (0.67-0.98) |
|  | Sex, Age,  Smoking level, %FVC | 217 | 0.71  (0.57-0.90) | 0.89  (0.71-1.09) |
| Non-IPF | None | 158 | 0.63  (0.49-0.82) | 0.83  (0.65-1.05) |
|  | Sex, Age,  Smoking level, %FVC | 156 | 0.73  (0.54-0.97) | 0.86  (0.66-.13) |

IPF, idiopathic pulmonary fibrosis; IIPs, idiopathic interstitial pneumonias; ESMI, erector spinae muscle index, PMI, pectoralis muscle index; OR, odds ratio; CI, confidence interval; FVC, forced vital capacity.
