# Supplementary figures and images for: Prognostic impact of muscle mass in idiopathic interstitial pneumonia: analysis of idiopathic pulmonary fibrosis and other idiopathic interstitial pneumonias
Source: BMC Pulm Med. 2025 Oct 14;25:468. doi: 10.1186/s12890-025-03942-0 (PMC12522827; doi:10.1186/s12890-025-03942-0)

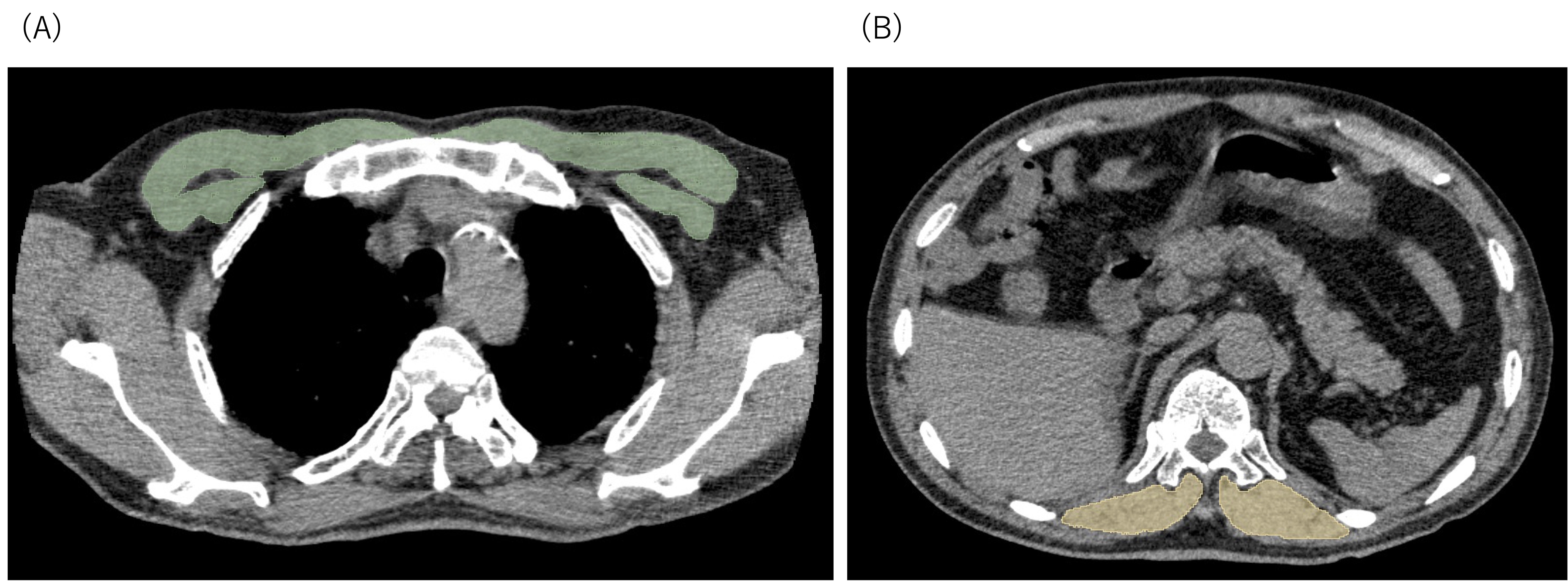

Supplement: Supplementary file 7 — Supplementary Material 7. Figure S1. Sample images of CT scans used for measurement of muscle areas pectoralis muscles cross section areas at T4 vertebra (A) and paraspinal muscles at T12 vertebra (B). [file 12890_2025_3942_MOESM7_ESM.jpg]

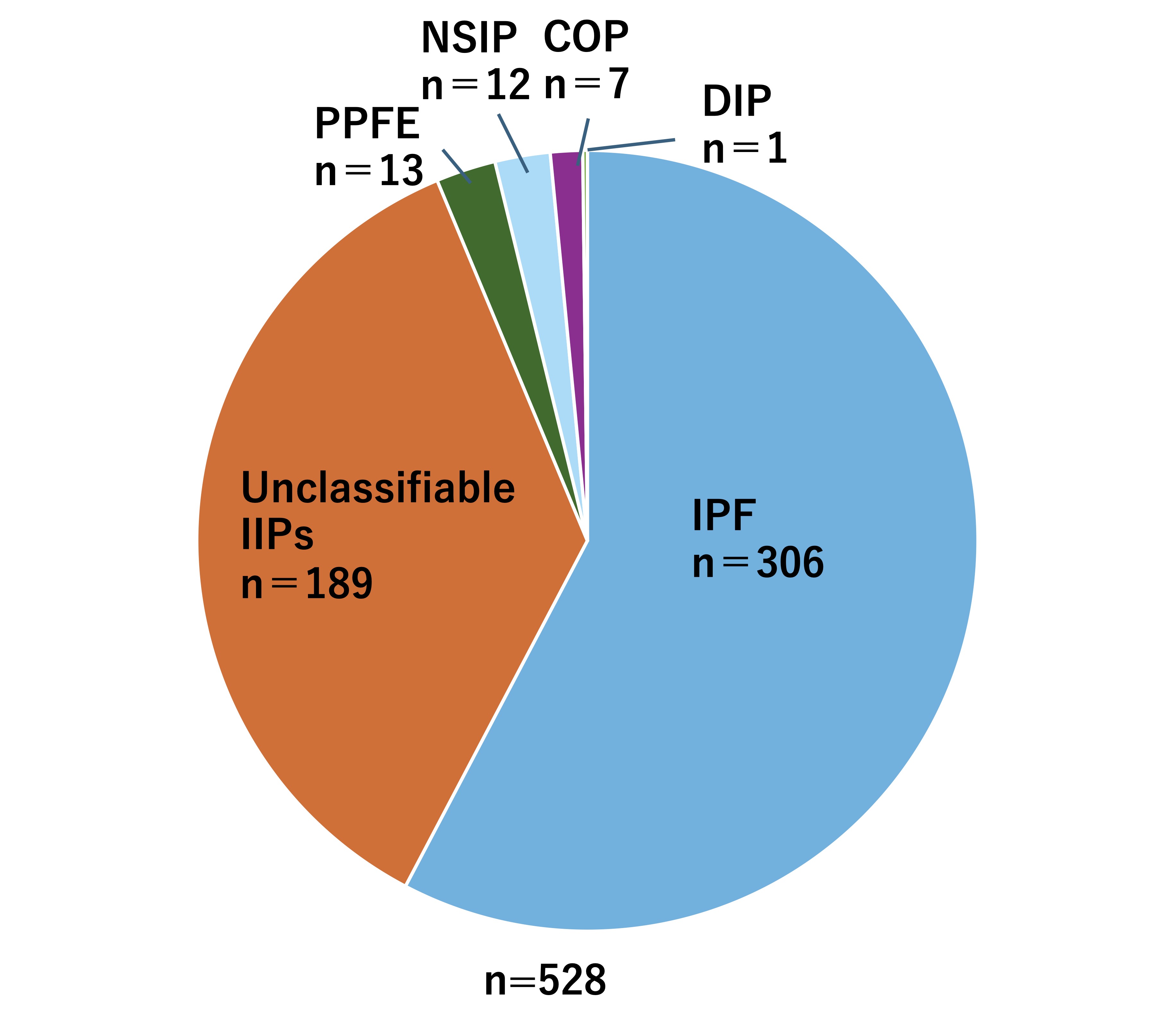

Supplement: Supplementary file 8 — Supplementary Material 8. Figure S2. Diagnosis for the study patients after multidisciplinary discussion. IPF, idiopathic pulmonary fibrosis; IIPs, idiopathic interstitial pneumonias. PPFE, pleuroparenchymal fibroelastosis; NSIP, nonspecific interstitial pneumonia; COP, cryptogenic organising pneumonia; DIP, desquamative interstitial pneumonia. [file 12890_2025_3942_MOESM8_ESM.jpg]

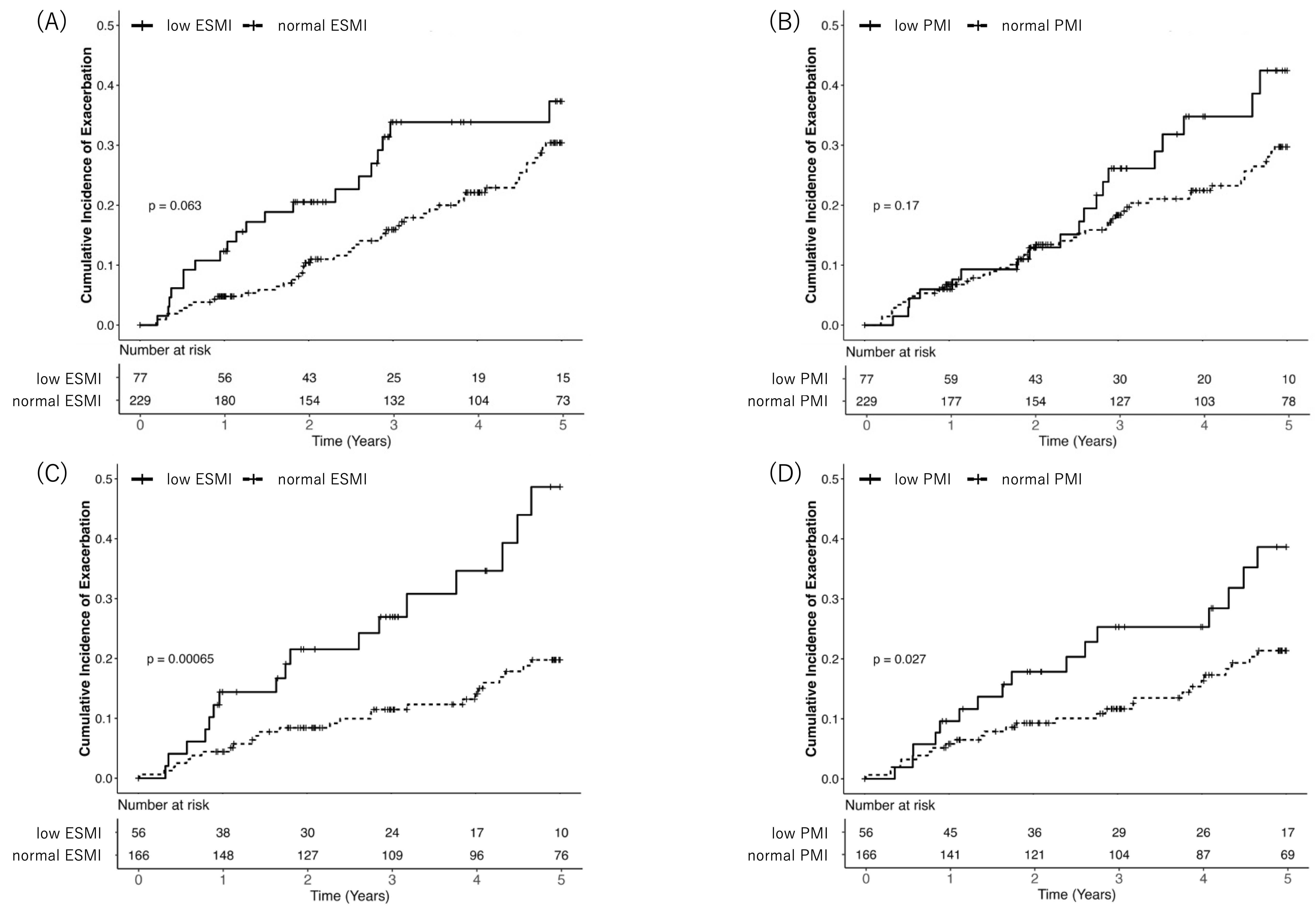

Supplement: Supplementary file 9 — Supplementary Material 9. Figure S3. Kaplan- Meier plot for the cumulative incidence of acute exacerbation stratified by ESMI or PMI groups.Kaplan- Meier curves plot for the cumulative incidence of acute exacerbation according to ESMI and PMI in patients with IPF (A, B) and non-IPF (C, D). IPF, idiopathic pulmonary fibrosis; ESMI, erector spinae muscle index; PMI, pectoralis muscle index. IPF, idiopathic pulmonary fibrosis. [file 12890_2025_3942_MOESM9_ESM.jpg]

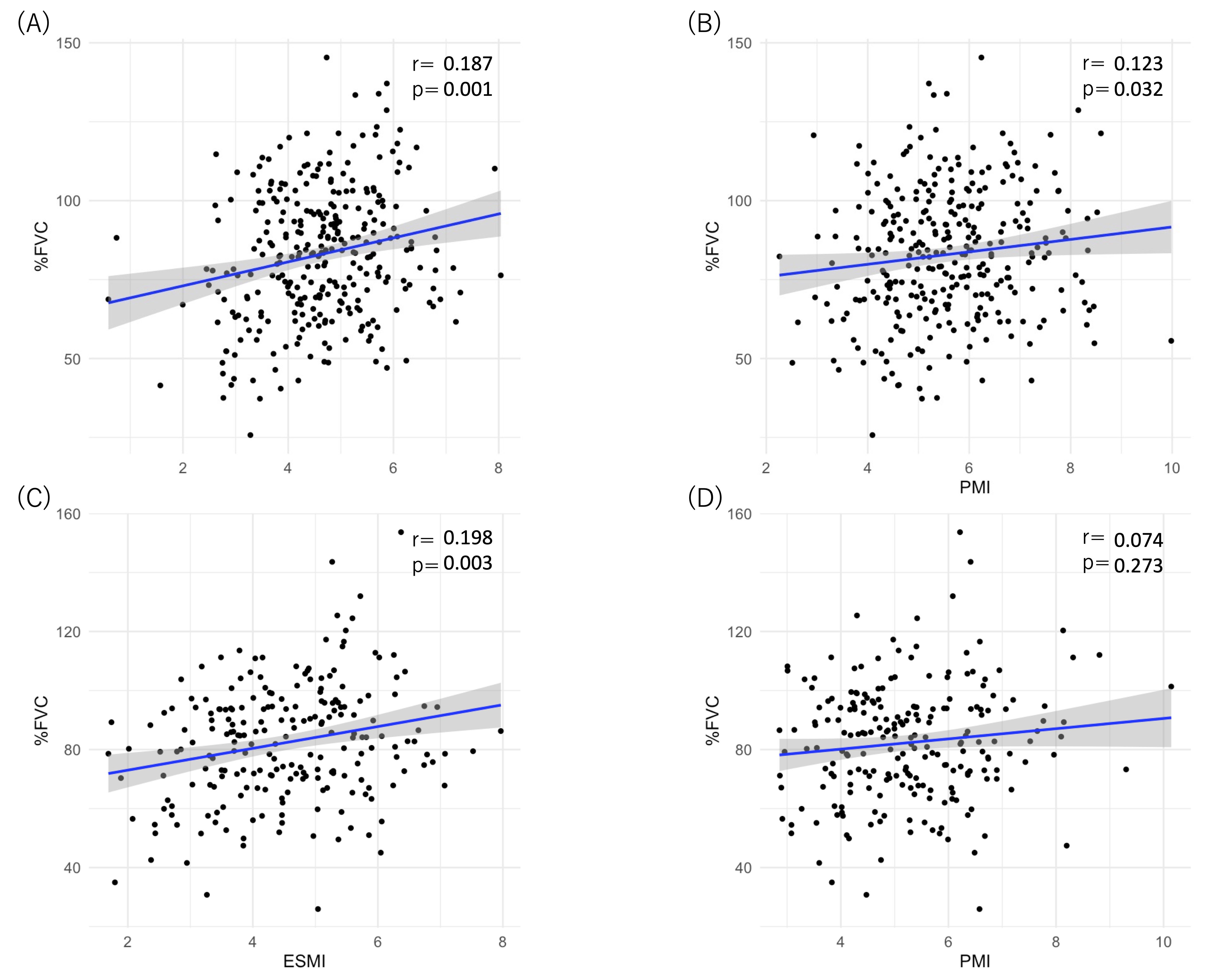

Supplement: Supplementary file 10 — Supplementary Material 10. Figure S4. Correlation between %FVC and ESMI or PMI according to IPF and non-IPF cohort. %FVC, forced vital capacity; ESMI, erector spinae muscle index; PMI, pectoralis muscle index. [file 12890_2025_3942_MOESM10_ESM.jpg]
